# A study protocol for developing a spatial vulnerability index for infectious diseases of poverty in the Caribbean region

**DOI:** 10.1080/16549716.2025.2461867

**Published:** 2025-02-11

**Authors:** Behzad Kiani, Beatris Mario Martin, Angela Cadavid Restrepo, Helen J. Mayfield, Eloise Skinner, Ana Karina Maldonado Alcaíno, Eric J. Nilles, Colleen L. Lau, Benn Sartorius

**Affiliations:** a UQ Centre for Clinical Research, Faculty of Health, Medicine and Behavioural Sciences, The University of Queensland, Brisbane, Australia; b School of Public Health, Faculty of Health, Medicine and Behavioural Sciences, The University of Queensland, Brisbane, Australia; c Department of Emergency Medicine, Brigham and Women Hospital, Boston, MA, USA; d Infectious Diseases and Epidemics Program, Harvard Medical School, Boston, MA, USA; e Infectious Diseases and Epidemics Program, Harvard Humanitarian Initiative, Cambridge, MA, USA

**Keywords:** Environmental factors, disease risk mapping, fuzzy analytic hierarchy process, seroprevalence data, risk factors, socioeconomic vulnerability, health accessibility, resource allocation, spatial analysis, climatic factors

## Abstract

Infectious diseases of poverty (IDoP) affect disproportionately resource-limited and marginalized populations, resulting in spatial patterns of vulnerability across various geographical areas. Currently, no spatial indices exist to quantify vulnerability to IDoP at a fine geographical level within countries, such as municipalities or provinces. Without such an index, policymakers cannot effectively allocate resources or target interventions in the most vulnerable areas. This protocol aims to specify a methodological approach to measure spatial variation in vulnerability to IDoP. We will evaluate this methodological approach using surveillance and seroprevalence data from the Dominican Republic (DR) as part of a broader effort to develop a regional index for the Caribbean region. The study will consist of three main components. The first component involves identifying the relevant factors associated with IDoP in the Caribbean region through a scoping review, supplemented by expert-elicited opinion. The second component will apply a Fuzzy Analytic Hierarchy Process to weigh the aforementioned factors and develop a spatial composite index, using open data and available national surveys in the DR. In the final component, we will evaluate and validate the index by analysing the prevalence of at least three IDoPs at a fine-grained municipal level in the DR, using seroprevalence data from a 2021 national field study and other national surveillance programs. The spatial vulnerability index framework developed in this study will assess the degree of vulnerability to IDoP across different geographical scales, depending on data availability in each country.

## Paper Context

- **Main findings**: The spatial vulnerability index identifies areas in the Dominican Republic at high risk of infectious diseases of poverty, integrating socio-economic, environmental, and healthcare factors.
- **Added knowledge**: This study demonstrates the importance of a geographically specific approach to assessing vulnerability, addressing limitations in existing poverty metrics for infectious disease risk.
- **Global health impact for policy and action**: The index provides a scalable tool for policymakers to target resources and interventions, enhancing disease prevention strategies in resource-constrained settings.

## Background

Infectious Diseases of Poverty (IDoP), such as malaria, tuberculosis, and neglected tropical diseases (e.g. lymphatic filariasis and dengue), are more prevalent in low- and middle-income countries [1]. These diseases disproportionately affect marginalized populations and are a result of socio-economic conditions, such as income, education, and employment, at both the individual and population levels. Additionally, environmental factors and limited access to healthcare – defined as physical accessibility, availability, and affordability – further exacerbate vulnerability to these diseases [2–5]. Despite the critical need to address the socio-economic and environmental determinants at a population level, there remains a lack of robust tools to accurately measure composite vulnerability to these diseases at a fine geographical scale, such as province or municipality, within countries.

Location-based socio-economic and environmental determinants play a critical role in shaping vulnerability to IDoP. For instance, poverty levels, population density, access to sanitary toilets, number of available healthcare facilities, and urbanization rates are socio-economic factors that influence community health outcomes [5–7]. In parallel, climatic conditions such as humidity, wind speed, precipitation, and temperature affect the transmission and prevalence of certain infectious diseases regardless of socio-economic status. For example, higher temperatures and precipitation can increase mosquito populations, leading to the spread of vector-borne diseases like Zika and lymphatic filariasis, while droughts and flooding can both increase transmission of diarrheal diseases [8]. These environmental factors apply broadly, but their impact can be more severe in economically disadvantaged areas where communities often lack the infrastructure or resources to mitigate disease risks. The interaction of these socio-economic and environmental factors creates complex vulnerability patterns at the population level. Without tools that incorporate spatial data, public health responses may overlook high-risk communities [9]. This oversight makes it challenging to allocate resources effectively and implement location-specific strategies, such as environmental sanitation improvements, healthcare accessibility initiatives, mass drug administration, tailored vaccination campaigns, or targeted public education efforts. Ultimately, these challenges hinder the ability to reach the goals of disease elimination programs [10,11].

The Caribbean region, with its distinctive combination of socio-economic and environmental factors, demonstrates the complex interplay of elements contributing to vulnerability to IDoP [12]. Within this region, climatic and environmental conditions facilitate the transmission of multiple IDoP, including vector-borne and water-borne pathogens. The transmission and control of these diseases are further exacerbated by socio-economic disparities, such as limited access to essential healthcare services and inadequate infrastructure [13]. These challenges highlight the urgent need for geographically specific tools that can accurately assess vulnerability to IDoP in the Caribbean. Such tools would not only enable the identification of current high-risk areas but also allow for the estimation of future risks by incorporating predictive models, such as those that assess the impact of different climate change scenarios [14,15]. This forward-looking approach would enhance the ability to proactively address endemic and emerging threats by adapting public health strategies to mitigate the long-term impact of IDoP in the region.

Multiple data sources are available to identify factors contributing to IDoP risk. Publicly available environmental data, such as those from the ECMWF Reanalysis 5th Generation (ERA5) or Climatic Research Unit Time-Series (CRU-TS) databases [16,17], along with national census data and specific surveys like the Demographic and Health Surveys (DHS), which include socioeconomic indicators, may provide insights into how these variables interact at the local level. Additionally, some studies have developed high-quality data that are particularly useful for this type of research. For example, Weiss *et al*. provided global maps of travel time to healthcare facilities, which can serve as a proxy for potential healthcare accessibility [18]. They also developed a global friction surface metric, which calculates travel time between locations by considering factors such as land elevation, landcover, infrastructure (e.g. paved and unpaved roads, railways), water bodies, and natural or political boundaries [19]. National disease-specific surveys offer valuable data, providing both predictors and outcomes for certain infectious diseases [20]. These surveys can be instrumental in evaluating a spatial vulnerability index, offering critical insights into how disease patterns correlate with socio-economic, environmental, and healthcare access factors across different regions. Finally, administrative data, such as access to electricity and waste collection, can provide valuable high-resolution information to enhance the development of the vulnerability index. Specific protocols, including obtaining aggregated and anonymized data, would be required for its use. Although obtaining such data involves uncertainties, its inclusion, if available, could significantly improve model performance.

While significant progress has been made in developing infectious disease vulnerability indices, most research has focused on specific groups of factors, such as social or climatic variables, and on individual diseases. For example, a social vulnerability index was developed to measure dengue vulnerability at the municipality level in Brazil [21], while a geo-environmental index in India assessed lymphatic filariasis transmission risk by identifying key variables such as temperature, relative humidity, rainfall, altitude, and soil texture as critical determinants of vector survival [22]. However, these indices are limited in scope as they focus solely on factors relevant to specific diseases, overlooking shared socio-economic and environmental drivers and missing opportunities to identify geographic hotspots where multiple IDoPs cluster or overlap. This narrow focus also limits the design of integrated public health interventions capable of addressing multiple pathogens simultaneously. A more comprehensive approach, considering multiple diseases and their unique transmission pathways, would offer a fuller understanding of public health vulnerabilities and enable more effective interventions. For instance, Moore *et al*. developed an infectious disease vulnerability index that incorporates a broad range of factors to highlight countries at higher risk of outbreaks [23]. While this provides a valuable global perspective, its focus on the country level makes it less suited for targeted interventions at finer geographical scales, such as municipalities or provinces, where tailored strategies are often more effective.

This study protocol outlines the development of a spatial vulnerability index for IDoP at the municipality level, specifically tailored to the Caribbean region. This localized approach may allow public health authorities to identify and prioritize high-risk areas more effectively, enabling the allocation of resources and implementation of interventions – such as vaccination campaigns, vector control programs, or sanitation improvements – where they are most needed. The index will be evaluated using data from a national serological survey in the Dominican Republic (DR) and the data of available national surveillance programs.

## Methods

**Figure 1.**
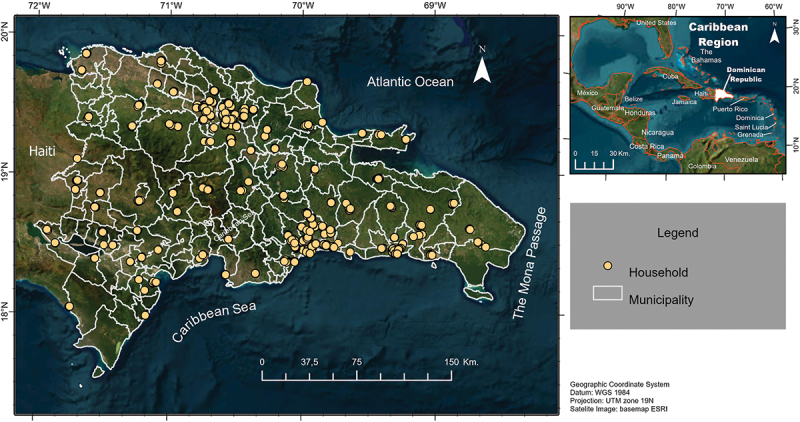
Map of the Caribbean region, with the Dominican Republic highlighted as the study focus. Yellow dots indicate approximate locations of household serosurvey sites from 2021, which will be used to evaluate the vulnerability index.

The Caribbean (Figure 1), home to diverse ecological zones and varying socio-economic contexts, presents a unique challenge for the control and prevention of IDoP. The region’s vulnerability is heightened by its geographic isolation as an island chain, combined with frequent exposure to hurricanes, flooding, and other natural disasters that facilitate disease outbreaks [24]. Economically, the Caribbean nations range from low- to upper-middle-income countries, with marked disparities in healthcare infrastructure and access to services. Culturally, the region’s history of migration and population movement influences disease transmission patterns, particularly for vector-borne diseases like dengue and lymphatic filariasis [25]. Moreover, the Caribbean is a focus area for multiple global disease elimination programs, including the WHO’s Global Leprosy Strategy and PAHO’s malaria elimination efforts, reflecting its strategic importance in global public health initiatives [26]. Latin America and the Caribbean account for one-third of global leptospirosis outbreaks [27], experienced a malaria outbreak in 2019 [26], and bear a significant burden of dengue and lymphatic filariasis [28,29].

The DR provides a unique opportunity to develop and test a spatial vulnerability index for IDoP. Located on the island of Hispaniola, the DR spans 48,442 km^2^ and has a tropical climate that supports a wide range of microclimates due to its diverse topography [30]. Average annual temperatures of 25°C, with two rainy seasons from April to June and September to November, create favourable conditions for vector proliferation and pathogen transmission [30,31]. The country’s geographic location also makes it highly susceptible to hurricanes and flooding, which exacerbate the spread of waterborne and vector-borne diseases, such as dengue, leptospirosis, and malaria [31,32].

Socio-economic disparities further compound vulnerability to infectious diseases. While the DR has seen economic growth and reductions in poverty over the past two decades, it remains one of the most unequal economies in the Caribbean [31,33]. Poverty is concentrated in rural areas and along the Haiti border, where healthcare access and infrastructure are limited [31]. The DR also reports frequent outbreaks of dengue and other mosquito-borne arboviruses, with variations in disease distribution influenced by climatic, geographic, and socio-economic factors [32,34]. These intersecting environmental and socio-economic challenges underscore the DR’s suitability as a location for developing a spatial vulnerability index tailored to the unique conditions of the Caribbean region.

This study will consist of three components. First, we will conduct a comprehensive literature review and engage an expert panel to identify relevant factors associated with various IDoP in the Caribbean region. Second, we will develop a spatial composite index that integrates and weighs these factors to measure vulnerability to IDoP at the municipality level in the DR. Finally, we will evaluate and validate this spatial index using national surveillance and serosurveillance data from the DR on selected IDoP, depending on data availability.

### Create a pool of relevant factors

#### Scoping review

We will include scientific databases such as PubMed, Web of Science, Scopus, and the Latin American and Caribbean Health Sciences Literature (LILACS). A systematic search strategy will then be implemented to retrieve all relevant articles from the Caribbean region that examine socioeconomic, environmental, or healthcare accessibility factors associated with IDoP. To develop this search strategy, we will first establish a comprehensive list of IDoP. The literature generally agrees that IDoP include neglected tropical diseases (NTDs), tuberculosis, HIV, and malaria, among others [35]. However, the specific diseases defined as NTDs have been subject to debate in recent years. We will use the most recent and comprehensive list of NTDs, including the additional diseases recently added to the World Health Organization (WHO) list [36]. An infectious disease specialist with experience in South America will review and refine the list of factors to ensure that only relevant IDoP are included. Diseases not prevalent in the Caribbean will be excluded during this process. The general search strategy includes a list of IDoP, terms reflecting concepts such as socioeconomic, environmental, and accessibility factors, and a list of Caribbean countries.

##### Selection of studies

###### Eligibility criteria

This study will include original research articles published in peer-reviewed journals that focus on the Caribbean region. Studies will be eligible for inclusion if they report the relationship between identified area-level risk factors and one or more burden metrics (e.g. prevalence, incidence, or outbreak) for any IDoP. Both quantitative methods (e.g. regression analysis, machine learning approaches, system dynamics) and qualitative methods (e.g. concept mapping) will be considered. Studies will be excluded if they examine associations at the individual level rather than the area level.

###### Screening process

One reviewer will initially screen the titles and abstracts of all studies. If there is any uncertainty about whether to include or exclude an article, the reviewer will consult with the team to reach a final decision. The full text of potentially relevant articles will then be reviewed to identify eligible studies for data extraction.

##### Data extraction and synthesis

We will review the included articles to compile a comprehensive list of factors associated with the IDoP burden. Additionally, we will extract the strength of these associations, where reported, and synthesize the findings to identify patterns, commonalities, and gaps in the literature. This synthesis will provide a clearer understanding of the key drivers of IDoP and guide the development of our spatial vulnerability index.

#### Refining the list of potential factors

In this component, we will use the Delphi method [37] to refine and validate the list of factors extracted from the literature. We aim to recruit 5–10 experts, ensuring representation for infectious diseases from different transmission groups (e.g. vector-borne, water-borne, and zoonotic diseases). Experts will be selected based on their expertise in infectious diseases, public health, and socio-economic and environmental factors affecting disease vulnerability. This group will include academics, local public health officials, clinicians, and representatives from WHO, CDC, and local organizations, with a focus on ensuring that key diseases such as malaria, lymphatic filariasis, and other IDoPs relevant to the Caribbean region, are appropriately represented.

Experts will be recruited through professional networks, collaborations with relevant organizations, and direct invitations. In the first round of the Delphi process, an online survey will be conducted, presenting the initial list of factors to the experts. They will review the list and suggest any additional factors they believe should be included, ensuring its comprehensiveness.

We will assess each unique factor to determine the availability of data specific to the DR. By focusing the scoping review on the broader Caribbean region, we aim to establish a comprehensive and data-rich foundation for the expert panel to refine and weigh the factors specifically for the DR. Additionally, by incorporating expert knowledge from various infectious disease transmission groups, we strive to address any gaps or biases in the literature. For instance, previous studies may have overemphasized certain diseases while underrepresenting others, leading to an incomplete understanding of the full range of risk factors for IDoP. Ensuring a balanced perspective helps make the final list of factors both comprehensive and representative of the diverse risks that may be present in the DR.

In the second round, participants will review a comprehensive list that includes the original factors and any additional factors suggested in the first round that have available data. Each factor will be accompanied by a brief summary of the supporting evidence. Additionally, we will refine the Delphi questions during the session to determine which specific measures are most appropriate for each factor (e.g. whether minimum, maximum, or average temperature should be used). Participants will vote on the inclusion of each factor using a Likert scale (e.g. strongly agree, agree, neutral, disagree, strongly disagree), and provide a certainty rating (e.g. very certain, moderately certain, neutral, slightly uncertain, very uncertain). To ensure accuracy across contexts, each factor’s relevancy and certainty rating will be recorded separately for each transmission group.

To determine inclusion, each factor must meet thresholds for both relevancy and certainty. Factors with high relevancy ratings (e.g. an average of at least 4 on the Likert scale) and a minimum average certainty rating (e.g. moderately certain or higher) will be prioritized for inclusion in the final list. Accordingly, the composite score threshold is set at 16, combining both relevancy and certainty ratings for each factor. This approach ensures that the final list reflects strong consensus and high confidence among experts. If any factors with empirical evidence from the literature fail to meet the threshold for inclusion based on expert votes, they will be excluded from the final list, maintaining alignment with the expert consensus.

Following the second round, a facilitated discussion will be conducted to clarify any differences in assumptions between the experts. This step is crucial for identifying factors that may differ between contexts, require further refinement or splitting, or where conflicting assumptions may exist [38]. The facilitated discussion will enable a deeper understanding of divergent opinions and ensure that the final factor list is contextually relevant and accurately reflects the expert consensus.

### Create a composite vulnerability index

We will identify and compile high-resolution data for key risk factors associated with IDoP in the DR. Geospatial analysis techniques will be applied to map and extrapolate the spatial distribution of these risk factors across municipalities.

When normalizing climatic risk factors such as precipitation and temperature, which vary continuously and non-linearly across scales and seasons, we acknowledge that simple normalization techniques (e.g. Min-Max scaling) might not fully capture their complex relationships with disease transmission. For instance, the relationship between precipitation and disease risk may peak at intermediate levels, while extreme values (too low or too high) could reduce risk. Similarly, temperature’s influence may be highly seasonal, requiring more dynamic modeling approaches.

To account for these complexities, we will explore advanced normalization techniques that better reflect the non-linear associations between environmental variables and disease risk. These may include:

- **Non-linear transformations** to adjust for thresholds or peaks in disease transmission (e.g. log transformations or polynomial scaling),
- **Cumulative or time-averaged measures** to reflect the build-up or decay of environmental risks over time.

We will evaluate the presence of multicollinearity among the selected variables. This will be done through correlation matrices or Variance Inflation Factor (VIF) analysis. In cases where variables are found to be highly correlated (e.g. land use, urbanisation, and forest cover), we will either exclude redundant variables or combine them into composite indicators. This will ensure that each factor contributes independently to the vulnerability index and avoids over-representation of any single factor in the model.

Next, we will employ the Fuzzy Analytic Hierarchy Process (Fuzzy AHP) to assign weights to the identified factors. To maintain consistency, we will engage the same expert panel from the Delphi technique. Participants will receive a brief literature review from step 1, highlighting a summary of these factors and their associations with IDoP outcomes.

Fuzzy AHP is particularly advantageous as it accommodates the uncertainty and imprecision inherent in expert judgments by utilizing fuzzy numbers rather than precise values in the pairwise comparison process. This approach enables experts to express their preferences more flexibly, allowing for degrees of agreement rather than forcing a rigid choice. Moreover, Fuzzy AHP improves the robustness of the weighting process, especially when dealing with complex and subjective criteria, such as socio-economic or environmental factors affecting IDoP. The use of fuzzy logic also helps mitigate subjective bias that can arise from variations in expert opinions, ensuring a more balanced assessment when determining the relative importance of each factor [39].

Finally, the weighted factors will be combined into a single composite index using a linear aggregation method, where each factor’s contribution to the index reflects its assigned weight, resulting in a spatial vulnerability map for IDoP.

### Evaluation

In this component, we will use data from a 2021 national serological survey in the DR to evaluate our constructed index against observed data for at least three IDoPs relevant to the region. The aforementioned survey aimed to characterize the epidemiology of a range of infectious diseases (including NTDs, vaccine-preventable diseases, vector-borne diseases, zoonotic diseases, and enteric diseases) in the DR and employed a spatial random sampling method to select clusters across the country, followed by nested household sampling. A total of 6,683 household members aged 5 years and older were enrolled in the national survey [40]. However, for diseases like tuberculosis and HIV, we will use notification data from the available national surveillance programs within the country.

We will evaluate how effectively the index reflects observed burden by applying various spatial analyses, including Moran’s I, Geary’s C, hotspot analysis, and geographically weighted regression (GWR). Specifically, we will assess whether the index corresponds with observed infection patterns, using the index as a predictor in spatial models to evaluate its relationship with disease burden. Metrics such as the degree of spatial autocorrelation (Moran’s I and Geary’s C) and the significance of spatial clusters (hotspot analysis) will be applied. For GWR, we will examine the strength of the relationship between the index and the selected IDoP burdens.

## Discussion

IDoPs exhibit distinct transmission dynamics and spatial vulnerability patterns compared to general infectious diseases [41], driven by the complex interaction of socio-economic, environmental, and healthcare access disparities, particularly in low-resource settings [5,42]. The spatial vulnerability index developed in the proposed study will be specifically tailored to the Caribbean context, where these factors are particularly relevant. However, the methodology will be adaptable to other countries or regions with similar IDoP epidemiology and related data. Applying the index outside the Caribbean would require careful consideration of regional differences in disease transmission and data availability to ensure its accuracy and relevance. The index can be constructed at different geographical scales based on the granularity of underlying data, allowing for tailored vulnerability assessments. In the DR, it will provide a high-resolution view at the municipality level, pinpointing areas most in need of urgent interventions.

The spatial vulnerability index accounts for the distinct transmission pathways of different IDoP groups, such as water-borne, vector-borne, soil-transmitted, and zoonotic diseases. Each of these disease categories is influenced by unique socio-economic and environmental factors. For instance, vector-borne diseases like Zika and lymphatic filariasis are closely linked to environmental conditions such as climate and land use [43], whereas zoonotic diseases like Leptospirosis are more directly affected by exposure to soil contaminated by the urine of infected animals, particularly following heavy rainfall or floods. This highlights the importance of clean water and sanitation infrastructure in mitigating such risks [44]. Additionally, the index will be flexible enough to incorporate the interactions between variables that may vary by context. For example, access to healthcare could differ in importance between rural and urban areas. In rural regions, healthcare access may be more critical due to facility scarcity, while factors such as population density or public transport availability may be more significant in urban settings.

By considering these interactions and context-specific factors, such as stratifying between rural and urban areas, the index can better reflect the complex, multifactorial drivers of disease transmission. Furthermore, incorporating models like GWR or interaction terms in the analysis will enhance the ability of the index to adjust weightings for different geographical settings [45]. The current design, which considers all IDoPs with the same transmission pathway together, enhances the understanding of shared spatial features and identifies hotspots where multiple diseases overlap, providing a comprehensive view of vulnerability. A data-driven approach – using methods such as machine learning or regression models – can further refine the index by generating objective weights tailored to each context. By customizing this approach for each disease and country, it is possible to accurately capture the interactions between socio-economic conditions, healthcare access, and environmental factors, enhancing both the precision and applicability of the index for guiding public health interventions.

To capture the variability within municipalities, we will account for differences in socio-economic conditions, healthcare access, and environmental risk factors by selecting appropriate measures such as the mean, median, or maximum values for each predictor, depending on the distribution and relevance of the data. This approach allows for the identification of areas most in need of intervention, guiding policymakers toward targeted, resource-efficient strategies. Additionally, the flexibility of the index could allow it to function as both a diagnostic tool and a potential predictive model. With reliable data inputs, the index could forecast future IDoP risks, facilitating a shift from reactive to proactive public health planning.

This study is likely to have some limitations. The availability and quality of data may be limited. To mitigate some of these challenges, satellite data can be integrated through platforms like Google Earth Engine which can process consistent, high-resolution environmental and human mobility data [46,47]. This will help fill gaps in ground-level data, and provide data on factors such as land use, vegetation, and climate patterns, which may impact the spatial dynamics of IDoP. Census data used for socio-economic indicators in the DR are not always complete or up to date. To overcome this limitation, we will utilise data from a large nationally representative survey conducted in the DR (*n* = 6683), which includes extensive data on SES factors at the household level [40]. Finally, while the fuzzy AHP method helps manage uncertainty, expert input is still subject to bias, which could influence the weightings assigned to various factors.

## Conclusion

The proposed spatial vulnerability index will offer a powerful tool for identifying and addressing vulnerability to IDoP across various geographical settings. Its flexibility to incorporate diverse data and expert insights will position it as a potential benchmark for precision public health interventions. With further validation, this index could be instrumental in predicting future IDoP risks based on key socio-economic and environmental factors, enabling proactive and targeted public health strategies. Ultimately, its application could enhance decision-making, optimize resource allocation, and support timely interventions in high-risk regions.
